# P-802. The Predictive Accuracy of Clinical Covariates in Differentiating *Staphylococcus aureus* Bacteremia with and without Deep-Seated Foci of Infection via Application of Different Variable Selection Methodologies

**DOI:** 10.1093/ofid/ofae631.994

**Published:** 2025-01-29

**Authors:** Amanda E Brunton, Luke Strnad

**Affiliations:** COPD Foundation, Exeter, New Hampshire; Oregon Health and Science University, Portland, Oregon

## Abstract

**Background:**

*Staphylococcus aureus* bacteremia (SAB) is a common bloodstream infection with a mortality rate of 20-30%. SAB is frequently associated with deep-seated foci of infection (DSFI) for which IDSA/ESCMID draft guidelines propose using clinical features early in evaluation to risk stratify for DSFI. However, literature for using combinations of clinical features to identify DSFI is limited, and the best process of variable selection is not defined. We explored variable selection methods to identify a combination of clinical features with the best predictive accuracy for identifying SAB with DSFI.

**Figure:**
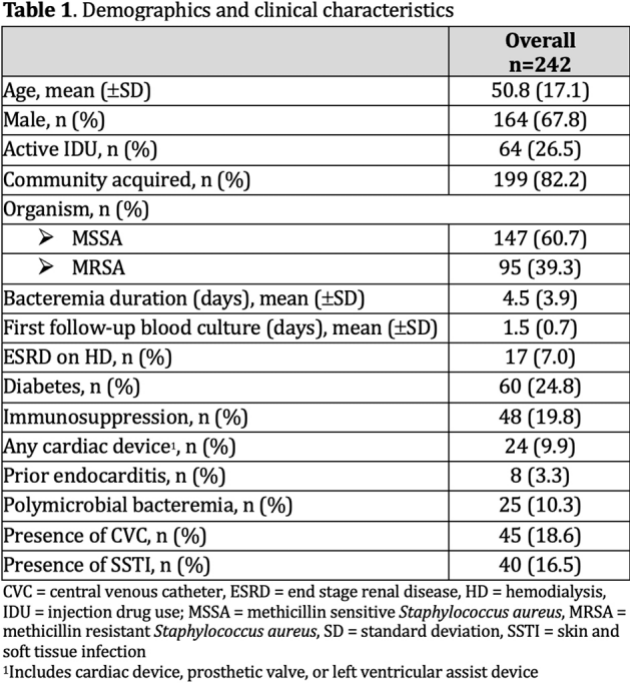

**Methods:**

242 adults admitted to a 500-bed teaching hospital in Portland, Oregon with incident SAB between January 1, 2016 and December 31, 2017 were included. Patients who died, transitioned to comfort or discharged before medically advised within 7 days, or without an infectious disease consultation were excluded. Clinical information was abstracted from electronic medical records. We performed four variable selection methods: best subset, forward and backward stepwise regression, and the LASSO. Variables were selected using 5-fold cross-validation. We estimated the area under the receiver operating curve (AUC) and fit a multivariable logistic regression model to evaluate the expected performance of the predictor combinations.

**Figure:**
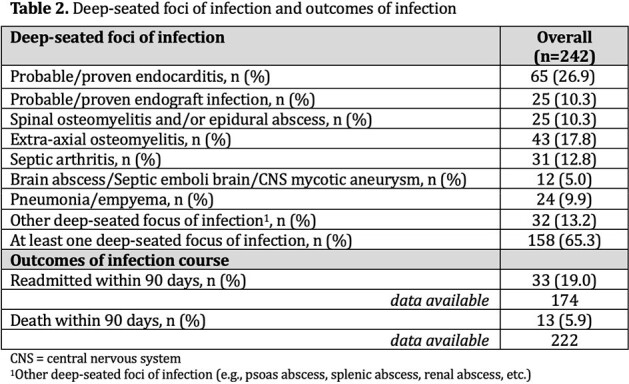

**Results:**

The cohort is described in Table 1 and 2. SAB with one or more DSFI was diagnosed in 65% (n=158). Predictors selected by the four methods are described in Table 3. The combination of bacteremia duration, hospital acquired SAB (haSAB), central venous catheter (CVC), skin and soft tissue infection (SSTI), and any cardiac device had the highest predictive accuracy with an AUC of 82%. The odds of DSFI is estimated to increase by 32% (95% CI; 15% to 53%) for every 1 day increase in bacteremia after adjusting for haSAB, CVC, SSTI and any cardiac device (Table 4).

**Figure:**
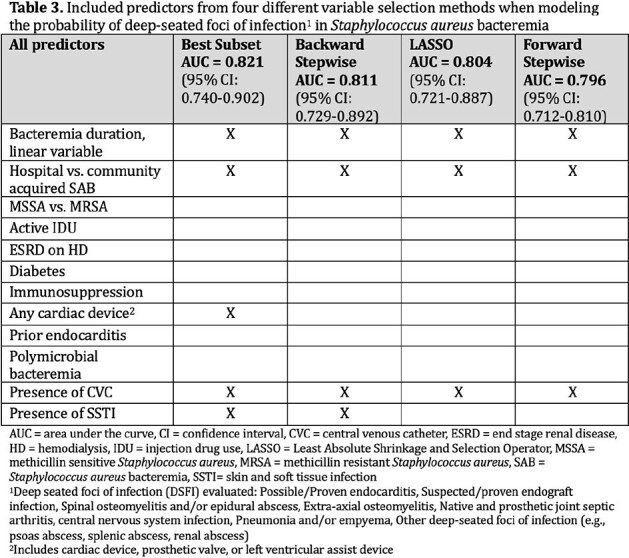

**Conclusion:**

Duration of bacteremia, haSAB, and CVC were included regardless of the variable selection method. Our model shows that combinations of clinical features are more predictive of DSFI than any single feature. Larger studies in different cohorts are needed to verify the predictive accuracy of these variables used in combinations to risk stratify for SAB with DSFI.

**Figure:**
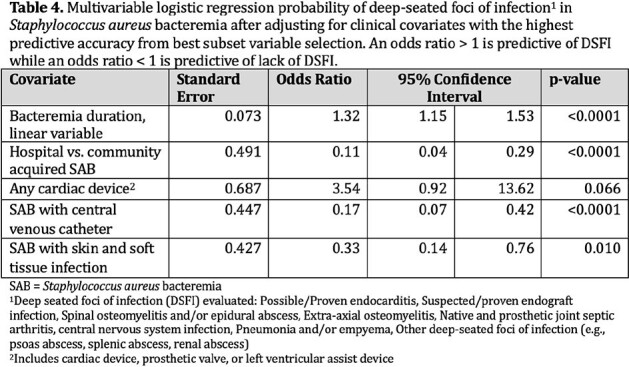

**Disclosures:**

**All Authors**: No reported disclosures

